# Coping strategies as modifiable pathways to posttraumatic growth in young adults facing parental cancer

**DOI:** 10.1017/S1478951525100448

**Published:** 2025-09-12

**Authors:** Erma Pratiwi Nufi, Alber Tigor Arifyanto, Minarti Usman, Wa Ode Lili Andriani Nasri, Ismail Ismail

**Affiliations:** 1 Guidance & Counseling Department, Universitas Halu Oleo, Kendari, Indonesia; 2 Biology Department, Universitas Halu Oleo, Kendari, Indonesia

## To the Editor,

We read with great interest the recent study examining the complex interplay between illness representations, coping strategies, and posttraumatic growth (PTG) in young adults whose parents have been diagnosed with cancer (Shinan-Altman and Becker 2024). This important work addresses a previously underrepresented demographic, offering valuable insights into the psychological processes that underpin resilience in the context of familial cancer.

Among the study’s many strengths, its identification of coping strategies as significant mediators in the pathway from illness perception to PTG stands out as particularly actionable (Leis et al. 2024). Coping strategies are not static traits but dynamic, modifiable behaviors that can be shaped through targeted psychosocial interventions (Crocetti et al., 2024). This presents a crucial opportunity for clinicians and mental health practitioners to develop structured interventions aimed at enhancing adaptive coping mechanisms in this population (Theodoratou and Argyrides 2024).

Interventions such as psychoeducation programs can play a pivotal role in reframing maladaptive illness perceptions and equipping individuals with constructive coping techniques (Vogelaar et al. 2024). Support groups, whether in-person or virtual, offer peer validation and shared coping resources, mitigating feelings of isolation and helplessness (Litvak Hirsch and Kassif Ben-Arie 2025). Additionally, the rising accessibility of digital mental health tools – including mobile applications and online cognitive-behavioral therapy modules – provides scalable, flexible platforms to deliver coping-focused interventions tailored to the unique challenges faced by young adults navigating parental cancer (Brotherdale et al. 2024).

By recognizing coping strategies as modifiable targets, psychosocial oncology services can shift from a reactive support model to a proactive resilience-building framework (Moravejosharieh and Dasht Bozorgi 2025; Paunescu et al. 2025). Early identification of maladaptive coping patterns and timely intervention not only promote PTG but may also reduce long-term psychological distress in this at-risk population (Dim et al. 2024).

We strongly advocate for future research and clinical initiatives to prioritize the development and systematic evaluation of coping-focused interventions for adult children of cancer patients. Integrating these strategies into family-centered oncology care models has the potential to transform the psychosocial outcomes for this overlooked group, fostering resilience and meaning-making in the face of profound familial adversity.
